# Exogenous 6-BA inhibited hypocotyl elongation under darkness in *Picea crassifolia* Kom revealed by transcriptome profiling

**DOI:** 10.3389/fpls.2023.1086879

**Published:** 2023-02-27

**Authors:** Hongmei Liu, Chengcheng Zhou, Zaib Un Nisa, Yousry A. El-Kassaby, Wei Li

**Affiliations:** ^1^ State Key Laboratory of Tree Genetics and Breeding, College of Biological Sciences and Technology, Beijing Forestry University, Beijing, China; ^2^ Cotton Research Institute, Multan, Punjab, Pakistan; ^3^ Department of Forest and Conservation Sciences, Faculty of Forestry, University of British Columbia, Vancouver, BC, Canada

**Keywords:** 6-BA, RNA-seq, transcriptome assembly, conifer, hypocotyl elongation

## Abstract

Hypocotyl elongation is an important process in plant growth and development, and is under hormonal regulatory signaling pathways. In our study, exogenous 6-BA significantly inhibited *Picea crassifolia* hypocotyl elongation more than ethylene in the dark, indicating the existence of different regulatory strategies in conifers, therefore, the *P. crassifolia* transcriptome was studied to explore the responsive genes and their regulatory pathways for exogenous N^6^-benzyladenine (6-BA) inhibition of hypocotyl elongation using RNA-Sequencing approach. We present the first transcriptome assembly of *P. crassifolia* obtained from 24.38 Gb clean data. With lowly-expressed and short contigs excluded, the assembly contains roughly 130,612 unigenes with an N50 length of 1,278 bp. Differential expression analysis found 3,629 differentially expressed genes (DEGs) and found that the differential expression fold of genes was mainly concentrated between 2 and 8 (1 ≤ log2FoldChange ≤ 3). Functional annotation showed that the GO term with the highest number of enriched genes (83 unigenes) was the shoot system development (GO: 0048367) and the KEGG category, plant hormone signal transduction (ko04075), was enriched 30 unigenes. Further analysis revealed that several cytokinin dehydrogenase genes (*PcCTD1*, *PcCTD3* and *PcCTD6*) catabolized cytokinins, while xyloglucan endotransglucosylase hydrolase gene (*PcXTH31*), WALLS ARE THIN 1-like gene (*PcWAT1-1*) and Small auxin-induced gene (*PcSAUR15*) were strongly repressed thus synergistically completing the inhibition of hypocotyl elongation in *P. crassifolia*. Besides, *PcbHLH149*, *PcMYB44* and *PcERF14* were predicted to be potential core TFs that may form a multi-layered regulatory network with the above proteins for the regulation of hypocotyl growth.

## Introduction

As a normal phenomenon, hypocotyl elongation is the result of long-term natural selection and a necessary prerequisite for photosynthesis and autotrophy of plants (Sliwinska et al., 2009). However, excessive hypocotyl elongation can easily cause seedlings to prematurely grow, making plants weak and susceptible to stress. Conifers are slow-growing, long-lived, and need to adapt to local environmental changes and weather extremes during their long-term lifespan (Farjon, 2018; Niu et al., 2022). Hypocotyl growth after seed germination and emergence has a large impact on conifer seedlings, which in turn affects recruitment and population distribution. *Picea crassifolia* is an important timber and ecological species in China (Wang et al., 2021). The natural range of this species is located in the dry zones of central and north-western China, and hypocotyl growth after germination will affect its survival, productivity and distribution range (Meng et al., 2007; Fan et al., 2016; Liu et al., 2016). Therefore, it is necessary to investigate the molecular mechanisms of *P. crassifolia* hypocotyl growth.

Seedlings hypocotyl growth is extremely plastic, with coordination between cell division and cell expansion to control hypocotyl length (Reed et al., 2018). This process is strongly influenced by external environmental conditions, such as light, temperature, humidity, gravity and touch, and these environmental signals eventually converge on phytohormones that regulate expansion of cells involved to achieve precise control of hypocotyl growth (Boron and Vissenberg, 2014; Song et al., 2019; Jiang et al., 2020). Therefore, hormonal signals are essential for hypocotyl growth. Among the various plant hormones, gibberellin, brassinolide and ethylene can significantly promote hypocotyl elongation, while external application of growth hormone such as cytokinin, jasmonic acid and abscisic acid can inhibit normal hypocotyl elongation to different degrees (Cary et al., 1995; Smalle et al., 1997; Collett et al., 2000; Lucas et al., 2008; Stamm and Kumar, 2013; Binder, 2020). In angiosperms, the molecular mechanisms of hormone signaling in hypocotyl elongation have been extensively studied (Cary et al., 1995; Smalle et al., 1997; Reed et al., 2018). For example, the transport inhibitor response 1 (TIR1) protein transmits IAA signaling to ARF transcription factors, which affect hypocotyl elongation by activating or repressing the target genes expression (Gray et al., 1998; Lucas et al., 2008; Oh et al., 2014; Nieto et al., 2015). GA affects the binding of DELLA protein to PIFs transcription factors by regulating its content, and PIFs transcription factors can contribute to the upregulation of the YUC8/9 expression, a key enzyme gene for auxin synthesis, to affect hypocotyl elongation (Feng et al., 2008; Lucas et al., 2008; Franklin et al., 2011; Mashiguchi et al., 2011; Lucas and Prat, 2014). Under light conditions, ethylene could promote hypocotyl elongation by activating phytochrome-interacting factor 3 (PIF3) through ethylene-insensitive 3 (EIN3), and under dark conditions ethylene could enhance the stability of ERF1 to inhibit hypocotyl elongation (Collett et al., 2000; Dan et al., 2003; Zhong et al., 2012). However, the mechanism of hormone regulation in hypocotyl growth varies between gymnosperms and angiosperms. The regulatory mechanism of hypocotyl elongation in conifers remains unclear.

Under light conditions, the regulation of hypocotyl growth by various hormones is dependent on the PIFs action, while under dark conditions hormones have different molecular mechanisms, and they can act independently or interact to promote or inhibit hypocotyl growth (Lucas et al., 2008; Franklin et al., 2011; Zhong et al., 2012). In addition, the significant promotion of hypocotyl elongation by hormones such as ethylene, auxin, BR and GA in angiosperms has been widely reported (Collett et al., 2000). However, in the present study, spraying exogenous 6-BA under darkness inhibited hypocotyl elongation in *P. crassifolia* showing similar effects as in angiosperms, the effect of 6-BA was significantly greater than that of ethylene, indicating that there may be a different 6-BA regulatory mechanism in gymnosperms from that of angiosperms. The present study was designed to explore the differences in hormone-regulated hypocotyl elongation between angiosperms and gymnosperms. We used transcriptomic data to identify the gene expression profiles and underlying the molecular mechanisms of the singling pathway under 6-BA-dark treatment in *P. crassifolia* by the RNA-sequencing.

## Materials and methods

### Plant material, treatments and sample collection

*Picea crassifolia* seeds used in this experiment were collected from trees growing in Zhangye City, Gansu Province. The seeds were randomly germinated in four plastic germination boxes under long-day conditions (16 h light/8 h dark) until germination. Seedlings were grown in controlled greenhouses with the daily average temperature of 25°C. The germination boxes were divided to two groups according to the provided light treatment. One group (one germination box) continued to grow under long-day, while the remaining three germination boxes were transferred to dark conditions. Under dark condition, one group (one germination box) received 100 ml water as control, and the other two germination boxes each received 100 ml water containing 80 μ mol L^-1^ of cytokinin (6-BA) and 450 μ mol L^-1^ of ethylene (ACC), respectively. After nine days of hormonal application hypocotyls were harvested and their length were measured using vernier calipers. Meanwhile, samples were quickly placed in liquid nitrogen and stored at -80°C for total RNA extraction and RNA-seq analysis. At same time, the same concentration (100 μ mol L^-1^) of ACC and 6-BA were used to treat the seedlings in the same way.

### Anatomical structure analysis

The hypocotyls with three replicates of each treatment were cut and fixed in FAA fixative solution. The fixed samples were dehydrated by ethanol with different concentration gradients from low to high. Then which were transferred to the mixture of 50% ethanol and 50% xylene for treatment for one hour, and then transferred to xylene for treatment for 40 minutes, and repeated twice. Removed xylene, add 2/3 xylene and 1/3 wax solution, and placed them in an oven at 42°C for two days. Two days later, replaced with a new pure wax solution and stewed overnight in an oven at 60°C. Replace the pure wax solution twice a day for 3 consecutive days. Then put the samples into plastic square boxs containing pure wax solution to embed the samples, and keep them still for one night. Properly trim the embedded samples, cut them into independent cubes, and store them at 4°C. Fix the samples on the paraffin microtome for sectioning, and set the slice thickness to 11nm. Take a rough look under the optical microscope, select the appropriate tissue and put it into the spreading machine for film development, and then place the glass slide on the 42°C drying machine for film drying. Took photos with fluorescence microscope.

### RNA isolation, library construction, and RNA sequencing

Total RNA from frozen hypocotyls with three replicates of each treatment were extracted by the Trizol method (Invitrogen, CA, USA). The final complementary DNA (cDNA) libraries were created using mRNA-Seq sample preparation kit. The cDNA libraries were sequenced on the BGISEQ platform by using the paired-end module (2 × 150 bp). The clean data analyzed during the present study are available in the NCBI-SRA Database (https://www.ncbi.nlm.nih.gov/sra/PRJNA895003).

### De-novo transcriptome assembly and transcript abundance estimation

De-novo assembly of clean data from all samples using Trinity (version 2.8.5, http://trinityrnaseq.sourceforge.net/). The first assembly results were clustered and de-redundant by cd-hit software (Li and Godzik, 2006) (version 4.8.1). The longest transcript of each gene was extracted from all transcripts using get_longest_isoform_seq_per_trinity_gene.pl script provided by Trinity as the Unigenes. Based on these Unigenes, we used align_and_estimate_abundance.pl script provided by Trinity to invoke the combination of RSEM (Li and Dewey, 2011) (version 1.3.3) and Bowtie2 (Langmead and Salzberg, 2012) (version 2.3.5) estimate the transcript abundance of all Unigene in each sample. The transcript abundance of all samples was used for the next step of differential analysis.

### Functional annotation and differential gene expression analysis

Homologous sequence of all unigenes was performed using BLASTx software (E-values ≤ 1.0 × 10^–5^) and their function annotations were searched against the GO, Nr, COG and KEGG databases. For other unannotated unigenes, we used the TransDecoder program (https://github.com/TransDecoder/TransDecoder) to predict their coding sequence (CDS) and orientation.

The transcript abundance of all unigenes from different samples was analyzed for differential expression using the R package DESeq2 (Version 1.24.0). Differences between treatment and control were evaluated by the form of fold changes, and this study took Log_2_foldchange ≥ 2 (*p* ≤ 0.01) and Log_2_foldchange ≤ -2 (*p* ≤ 0.01) as a criterion for screening upregulated and downregulated DEGs. Gene expression patterns were calculated and standardized using Z-scores transformation (Cheadle et al., 2003). Heatmap of DEGs expression patterns was conducted using the R package pheatmap (version 1.0.12).

### Gene regulatory network analysis

The Cytoscape CytoNCA tool (Tang et al., 2015) was used to analyze analyzed all of the top 10 differentially expressed hub genes. We performed the co-expression network using R package igraph (Ju et al., 2016) based on the Pearson correlation coefficient between hub genes and related genes.

### The qPCR validation

To validate the RNA-Seq data, core transcription factors and downstream proteins with up- or down-regulation during the 6-BA-Dark treatment were selected to perform qRT-PCR validation. The hypocotyls with six replicates of dark treatment and 6-BA-Dark treatment were collected and immediately placed in liquid nitrogen and stored at −80°C. Primers designed for qRT-PCR are given in **Table 1**.

**Table 1:** Differentially expressed genes selected for gene expression analysis by qRT-PCR.

No	Gene ID	Gene function	Primer Sequence
1	*PcbHLH149*	bHLH family transcription factor	F: AAAGGAATACAAGGCCGCCA
			R: CGTCCACAAGACCTGTCGAA
2	*PcMYB44*	MYB family transcription factor	F: CCCTTGACTGCCTTCCTCTG
			R: TCGTTAATGCAGGCGTCACT
3	*PcERF14*	AP2/ERF family transcription factor	F: CGTTCGGATGAGGAGATGGG
			R: CGGAGAGCGGAGTCAAATCA
4	*PcCTD1*	Cytokinin dehydrogenase 1-like	F: CCGCTGTTGGTTTGGTAACG
			R: CCTTTCCCTGTGACGACCTC
5	*PcCTD3*	Cytokinin dehydrogenase 3-like	F: ACGTCCTGCAACTCGACATT
			R: CATGTTCAGCCATGGATGCG
6	*PcCTD6*	Cytokinin dehydrogenase 6-like	F: TGGATTTTGGATGCTCGCCT
			R: TACGTCCCCTTTCCCTGTCA
7	*PcXTH31*	Xyloglucan endotransglucosylase hydrolase 31-like	F: GGGGATTGTGACGACGTTCT
			R: TCGGTGGAGAGTGTAGGAGG
8	*PcWAT1-1*	WAT1-related 1-like	F: GCTGATGACGTTATACAAAGGTCC
			R: GATGTCCCATCCTAAAGCCCA
9	*PcSAUR15*	Small auxin-induced 15A-like	F: AGGAGCAAGTTTCAGAGGCT
			R: TGTGAATACCGTCCGTGCTT

## Results

### 6-BA inhibit hypocotyl elongation than ACC under darkness in *P. crassifolia* seedlings

**Figure 1:**
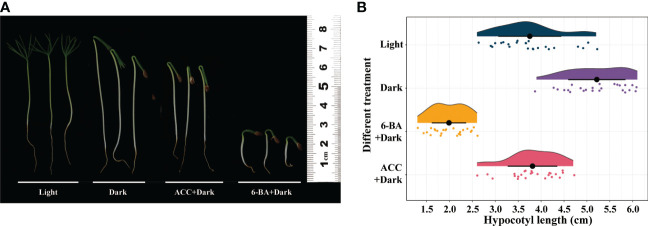
Effect of light, dark, ACC+Dark, and 6-BA+Dark on hypocotyl elongation in *P. crassifolia* seedlings. **(A)** Hypocotyl phenotypes of P.crassifolia under different treatments. **(B)** Cloud and rain plots of hypocotyl length statistics of P.crassifolia under different treatments.

We examined the effects of light, dark, 1-aminocy-clopropane-1-carboxylic acid (ACC), and 6-BA on the regulation of hypocotyl elongation in *P. crassifolia* germinants. ACC and 6-BA irrigation caused an inhibit effect on hypocotyl elongation, while conversely the dark condition caused a promotion effect (**Figure 1A**). 6-BA significantly inhibited seedlings hypocotyl elongation under control conditions after 2 weeks, and the average length of hypocotyl was 1.988 cm (**Figure 1B**). ACC has only a slight inhibit effect on hypocotyl growth.

**Figure 2:**
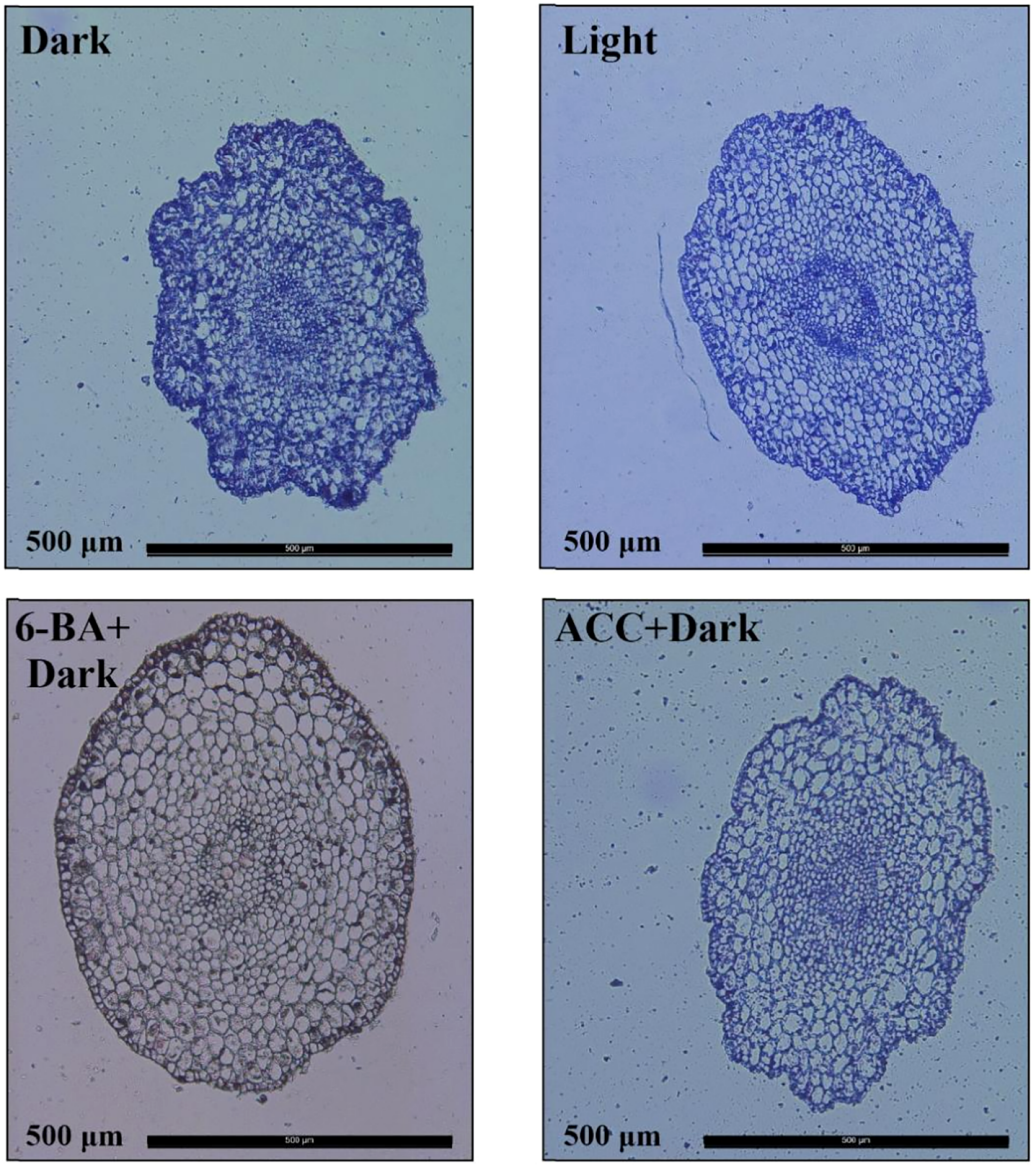
Cross-sectional cellular anatomy of *P. crassifolia* hypocotyls under light, dark, ACC+Dark, and 6-BA+Dark treatments.

To investigate how 6-BA treatment inhibited hypocotyl elongation in spruce, we observed the cross sections of hypocotyls of the four treatments by histological sections and found that the number of hypocotyl cells under 6-BA-dark treatment was significantly less than the other three treatments (**Figure 2**), indicating that the application of exogenous 6-BA inhibited the cell division of hypocotyl. Although the optimal treatment concentration of different phytohormones is different, for the sake of experimental rigor, we still explored the effects of 6-BA and ACC on the hypocotyl at the same concentration (100 μ mol L^-1^). The results showed that 6-BA-Dark treatment also produced a more pronounced inhibitory effect than ACC-Dark treatment at the same concentration (**Figure S1**).

### Sequencing analysis and *de novo* assembly of dark and dark-6-BA treatments

To study the molecular mechanisms of hypocotyl inhibit effect under 6-BA-dark treatment, we sequenced the six samples from 6-BA-dark and dark treatments. After filtering out adapter sequences and reads ≤ 50 bp, 117,221,270, and 126,553,322 clean data were obtained from samples in the dark and 6-BA-dark treatments, respectively (**Table 2**). A total of 130,612 unigenes (mean length 897 bp, N50 length 1287 bp) were identified from the above clean data. The GC content of all unigenes was 42.03%. Analysis of length distribution demonstrated that 23.43% of the unigenes were >1 kb.

**Table 2:** Summary of Illumina sequencing and mapping of *P. crassifolia* hypocotyls.

	Dark	6-BA+Dark
Number of raw reads	123,902,720	134,036,862
Number of clean reads	117,221,270	126,553,322
Q30 (%)	93.02	92.92
GC content (%)	45.83	45.81
Mapped on reference (%)	83.61	84.32
Multi-mapped (%)	3.29	3.46
Non-splice reads (%)	54.34	54.67
Splice reads (%)	25.98	26.39

### Differential gene expression analysis and functional annotation between dark and dark-6-BA treatment

**Figure 3:**
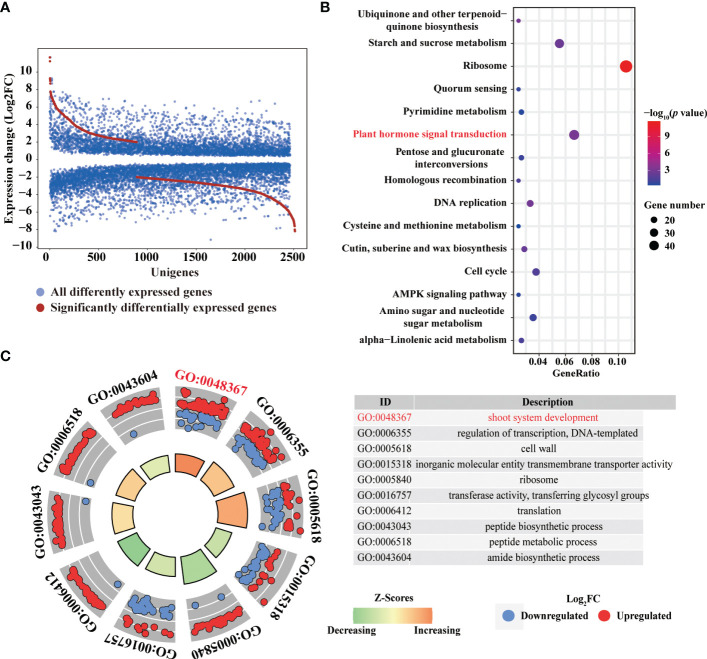
Transcriptomic changes in response to 6-BA in *P. crassifolia* hypocotyls. **(A)** The expression profiles of all DEGs. **(B)** The top 15 pathways in KEGG. **(C)** The top 10 categories in GO.

**Figure 4:**
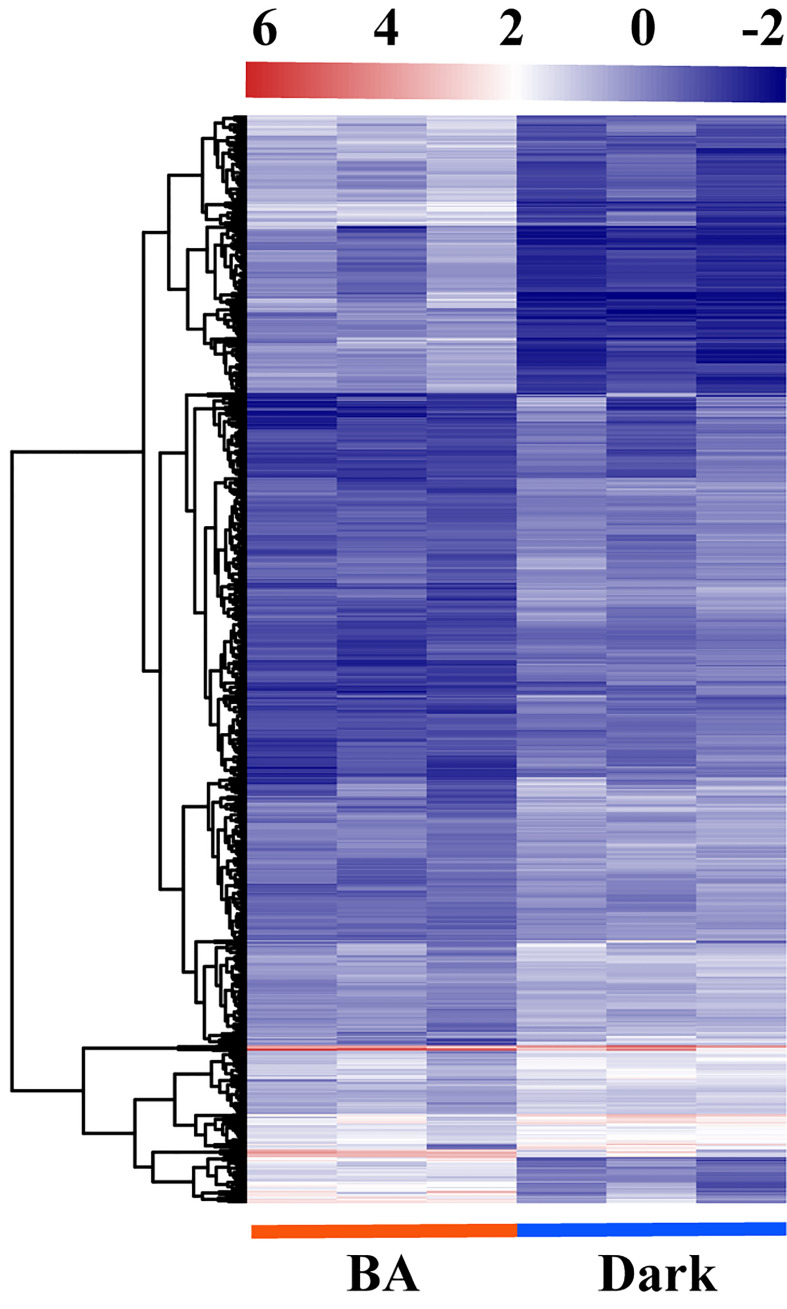
Transcriptomic changes of DEGs in response to 6-BA in *P. crassifolia* hypocotyls.

To identify essential genes and pathways involved in 6-BA treatment, we analyzed glo6-BAl gene expression in response to 6-BA and dark treatment. We identified 3,629 differentially expressed genes (DEGs) and found that the differential expression fold of genes was mainly concentrated between 2 and 8 (1 ≤ log_2_FoldChange ≤ 3; **Figure 3A**), indicating that 6-BA treatment caused a substantial change in gene expression compared with dark treatment. Meanwhile, we used a high-sensitivity threshold (log_2_FoldChange ≥ 2, *P* ≤ 0.01) for screening significantly differentially expressed genes and identified 807 significantly differentially expressed genes, accounting for 22.24% of all DEGs. A total of 559 unigenes (69.27% of all significant DEGs) were down-regulated by 6-BA treatment compared to the dark treatment (**Figure 3A**), indicating that the expression of a large number of genes were significantly repressed in *P. crassifolia* seedlings treated with exogenous 6-BA. Meanwhile, the expression pattern of 807 significant DEGs also confirmed this observation (**Figure 4**).

To obtain the main biological function and pathway of all significantly DEGs, gene ontology and pathway annotation were performed using OmicsBox and Panther (Thomas et al., 2003) respectively. A total of 807 significantly DEGs were annotated in 184 GO terms, including 114 biological processes (BP), 55 molecular function (MF) and 15 cellular component (CC). We selected the top 10 GO terms with enrichment numbers other than DNA binding terms for further analysis (**Figure 3C**) and found that the GO term with the highest number of enriched genes (83 genes) was shoot system development (GO: 0048367). Similarly, GO tems cell wall (GO: 005618) also contained 55 unigenes, these results indicated that a large number of genes regulated by 6-BA treatment are involved in the growth and elongation of cells in the shoot thus regulating the development of seedling hypocotyl.

KEGG pathway enrichment analysis of all DEGs was performed to characterize the complex biological behaviors. The enriched pathways reflected the preferential biological functions of samples from 6-BA and dark treatment. All significantly DEGs were annotated in 136 KEGG categories and we selected the top 10 KEGG catagories with enrichment numbers for further analysis (**Figure 3B**). Of KEGG pathways, most unigenes were assigned to “Ribosome” (49 unigenes, ko03010), “Plant hormone signal transduction” (30 unigenes, ko04075), and “Starch and sucrose metabolism” (25 unigenes, ko00500). The discovery of these genes related to plant hormone signal transduction indicated that several hormone signal transduction pathways were activated by 6-BA treatment and these hormone signals further activated downstream genes related to cells growth and elongation. These genes involved in hormone signal transduction pathways may help elucidating the molecular mechanisms underlying primary regulation network of hypocotyl elongation in the 6-BA treatment.

### Differential expression of transcription factors, key proteins involved in 6-BA treatment

**Figure 5:**
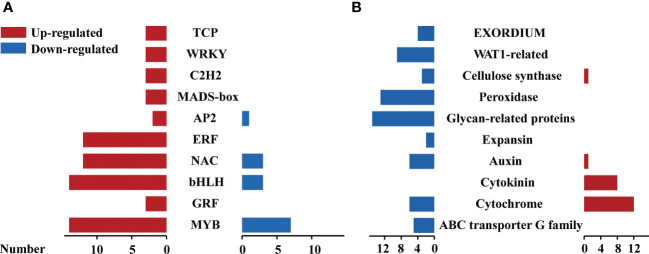
Profiles of transcription factors, key proteins in response to 6-BA treatment in *P. crassifolia* hypocotyls. **(A)** Differentially expressed transcription factors against 6-BA treatment. **(B)** Differentially expressed key proteins against 6-BA treatment.

According to the annotation of all unigenes, we found that a total of 110 unigenes were classified as transcription factors (TFs) in 807 significant DEGs. These TFs are comprised of 26 families including MYB, AP2/ERF-ERF, bHLH and NAC were detected and 91 of them were up-regulated by 6-BA treatment, indicating that the expression of several TFs were activated to regulate the expression of downstream genes. The regulated gene number of MYB family was highest among all TFs families under 6-BA treatment (**Figure 5A**), MYB38 was the highest up-regulated expression gene among all recorded MYB TFs and MYB6 that showed significantly increased expression in response to 6-BA-dark treatment. Transcriptomic data showed that 21 MYB genes were significantly activated under 6-BA treatment out of which 67% and 33% genes were up-regulated and down-regulated, respectively. In *Arabidopsis*, MYB transcription factor genes like MYBH, is one of the molecular components that influence hypocotyl elongation under darkness (Kwon et al., 2013).

When the plant senses external environmental changes, it activates downstream functional proteins to trigger corresponding physiological and biochemical changes through various levels of signal transduction. In the present study, 22 protein categories were mainly identified by functional annotation in 697 unigenes other than 110 TFs, including cytochrome-related proteins, ABC transporter G family proteins, cytokinin-related proteins, auxin-related proteins, WALLS ARE THIN1-related (WAT1) proteins, EXORDIUM-related proteins, and expansin-related proteins. In contrast to the expression pattern of transcription factors, the gene expression of downstream functional proteins was overwhelmingly down-regulated and a few were up-regulated under 6-BA treatment (**Figure 5B**). In our study, we found that 8 cytokinin-related proteins included 7 cytokinin dehydrogenase and a cytokinin hydroxylase, the 7 cytokinin dehydrogenase unigenes were ranked in the top 30 of all DEGs with high upregulation expression. Cytokinin dehydrogenase is the key enzyme of the cytokinin degradation pathway.

### Identification of hub genes associated with 6-BA-mediated inhibition of hypocotyl elongation in *P. crassifolia* seedlings

**Figure 6:**
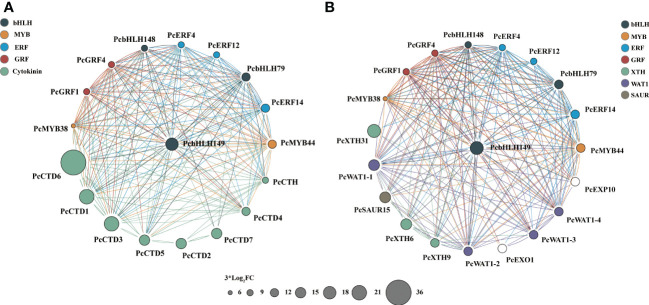
DEGs generate TFs network through Cytoscape ClueGo in *P. crassifolia* hypocotyls. **(A)** The co-expressed network between 10 core TFs and Cytokinin-related genes. **(B)** The co-expressed network between 10 core TFs and other key genes.

**Figure 7:**
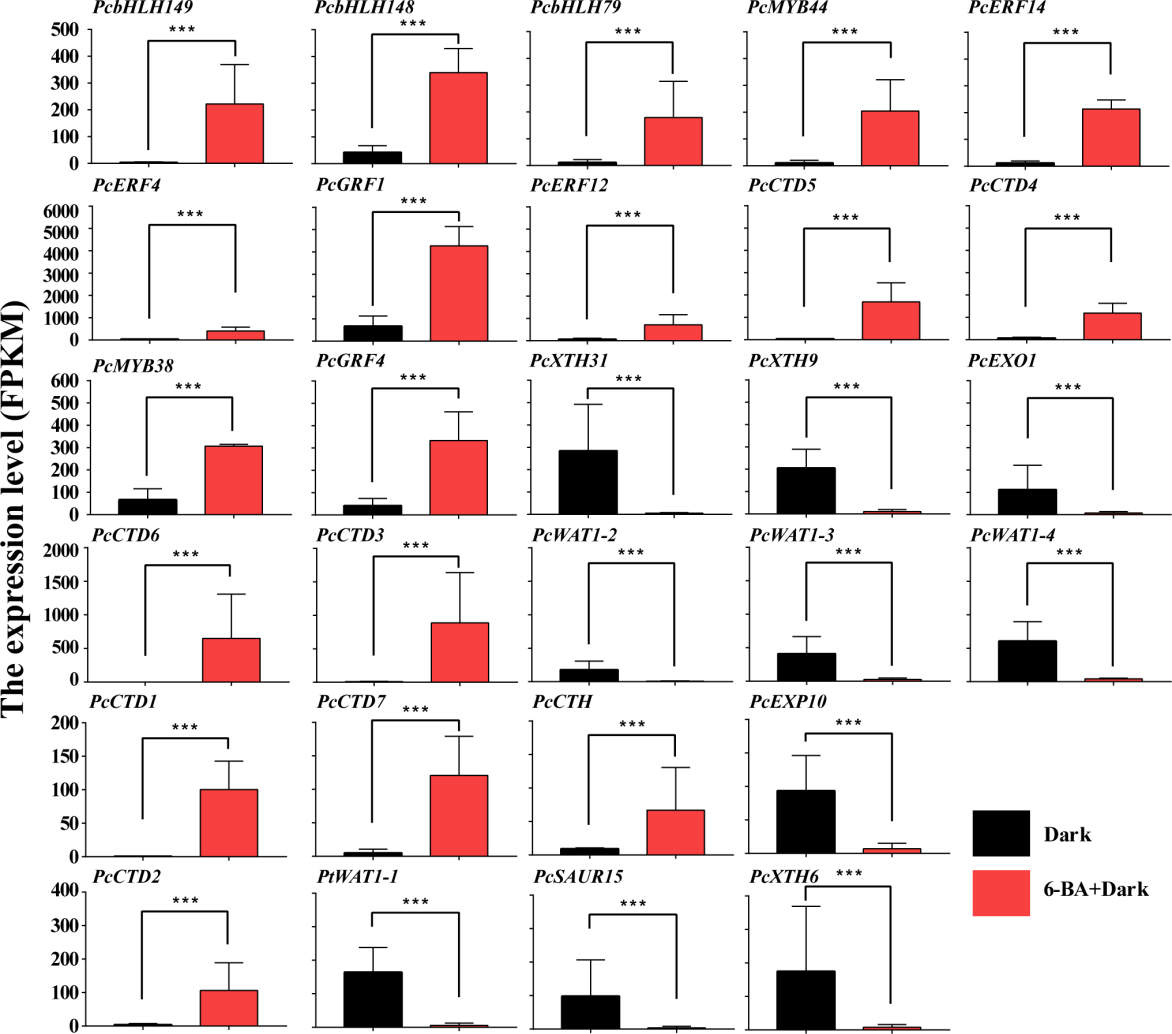
The gene expression profile of key TFs and proteins. ***: highly significant difference.

After analysis of different key transcription factor families, we screened for collaborative 10 hub up-regulated TFs, 8 up-regulated unigenes and 10 down-regulated unigenes associated with 6-BA treatment. We constructed two gene co-expressed network based on gene expression data and Pearson correlation among genes (**Figures 6**, **7**). In total, 28 genes were recorded in all samples, and they may have a critical role in the 6-BA-mediated hypocotyl growth inhibition.

All key genes in the co-expressed network were differentially expressed. Among 10 core TFs, 2 belong to MYB families (*PcMYB38* and *PcMYB44*), and 3 belong to ERF families (*PcERF4*, *PcERF12*, and *PcERF14*), and 2 belong to GRF families (*PcGRF1* and *PcGRF4*), and 3 belong to bHLH families (*PcbHLH79*, *PcbHLH148* and *PcbHLH149*). The *PcbHLH149* is highly induced by 6-BA treatment and plays a central role in two co-expressed networks. The interaction of BHLH with other proteins is dependent on their conserved motifs and their domain and hence the capacity to assemble into distinct TF complexes to modulate and/or empower their activity and/or specific function (Feller et al., 2011; Sun et al., 2018; Hao et al., 2021). Their functions are reported against several abiotic and phytohormone stresses, such as cytokinin, JA, GA and others. These hub up-regulated TFs are potential genes to activate or inhabit the expression of downstream key genes.

Among the 8 upregulated key genes, there were seven cytokinin dehydrogenases and one cytokinin hydroxylase, which are all cytokinin-related genes. *PcCTD6* expression level was significantly induced and up-regulated nearly 700-fold under 6-BA treatment. The cytokinin dehydrogenase, a flavinase that irreversibly degrades cytokinin to adenine/adenosine, is essential for maintaining cytokinin homeostasis in plants. This likely indicates that cytokinin dehydrogenase is used to regulate endogenous and exogenous cytokinin levels in *P. crassifolia* after receiving exogenous cytokinin signals. On the other hand, 10 down-regulated key genes are primarily associated with cell elongation, including xyloglucan endotransglucosylase hydrolase (XTH, including *PcXTH6*, *PcXTH9* and *PcXTH31*), WALLS ARE THIN 1 (WAT1, including *PcWAT1-1*, *PcWAT1-3*, and *PcWAT1-4*), Small Auxin Up-Regulated (SAUR, including *PcSAUR15*), and expansin (EXP, including *PcEXP10*) genes. These genes belong to the key genes in the acidic growth theory model and are mainly regulated by auxin, suggesting that the exogenous cytokinin treatment also couples with the auxin regulatory pathway thereby regulating hypocotyl growth.

### Validation of transcripts by qRT-PCR

**Figure 8:**
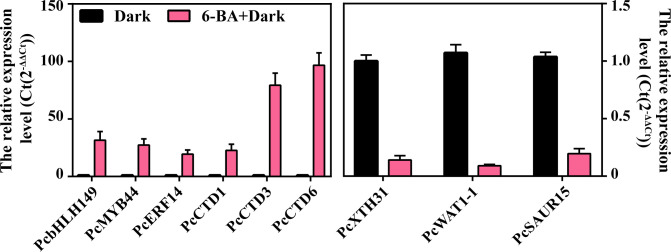
The qRT-PCR validation of PcbHLH149, PcMYB44, PcERF14, PcCTD1, PcCTD3, PcCTD6, PcXTH31, PcWAT1-1 and PcSAUR15.

The top 3 genes in terms of differential fold were selected to validate the RNA-seq data from key transcription factors, up-regulated genes and down-regulated genes, respectively. A total of 9 genes were used to perform qRT-PCR validation (**Table 1**), including *PcbHLH149*, *PcMYB44*, *PcERF14*, *PcCTD1*, *PcCTD3*, *PcCTD6*, *PcXTH31*, *PcWAT1-1* and *PcSAUR15*. Among these genes, *PcbHLH149*, *PcCTD6* and *PcXTH31* showed higher expression under 6-BA treatment (**Figure 8**). The validation results illustrated the reliability of the transcriptome data.

## Discussion

Conifers has been around for at least 300 million years and are able to adapt and diversify (Farjon, 2018; Niu et al., 2022). As the first event in plant growth that influences its survival and distribution, the regulation of hypocotyl elongation has an important role in their long-term evolution and adaptation to the environment (Ince and Galvão, 2021). Hypocotyl elongation is very plastic and is strongly influenced by factors that regulate cell elongation such as light, plant hormones, temperature, and touch (Reed et al., 2018). Different plant hormones are able to regulate hypocotyl elongation not only individually but also through interactions. In angiosperms, gibberellin, brassinosteroids and ethylene significantly promoted hypocotyl growth, while external application of growth hormone such as cytokinin, jasmonic acid and abscisic acid inhibited normal hypocotyl growth to varying degrees (Cary et al., 1995; Smalle et al., 1997; Collett et al., 2000; Lucas et al., 2008; Stamm and Kumar, 2013; Binder, 2020). The physiological phenomena of the hypocotyls regulation by these hormones are common in angiosperms and the molecular mechanisms have become increasingly clear, but the physiological phenomena and molecular mechanisms in conifers remain unclear. Additionally, the regulatory hormones and regulatory patterns of hypocotyl growth are different throughout the day (e.g., during the day and night), for example, ethylene promotes and inhibited hypocotyl elongation in the light and darkness (Yu et al., 2013). Cytokinin inhibits hypocotyl elongation in darkness but has no obvious effect in the light (Smets et al., 2005). During the day when nighttime temperatures are low, hypocotyl overgrowth can expose itself to damage from cold temperatures (Seo and Yoon, 2019; Liu et al., 2021). Therefore, the regulation of plant hypocotyls growth under dark conditions is more important to study. In this study, the *P. crassifolia* hypocotyl growth was investigated using four different treatment conditions: light, darkness, ACC under darkness, and 6-BA under darkness. We found that the inhibitory effect of ethylene on hypocotyl was not significant under dark condition, while that of cytokinin was very significant. This suggests that, under darkness, cytokinin has a greater regulatory effect on hypocotyl than ethylene in gymnosperms.

To explore the molecular mechanism of cytokinin-inhibited hypocotyl elongation in *P. crassifolia*, the present study evaluated the RNA-seq data of *P. crassifolia* hypocotyls between darkness and 6-BA under darkness conditions. We identified 807 significantly differentially expressed genes, the expression level of 69.27% of all significant DEGs were downregulated by 6-BA treatment under darkness, suggesting that the inhibition of hypocotyl elongation in *P. crassifolia* may be caused by the suppression of a large number of cell growth genes. However, transcription factors (TFs) have a pivotal role in responding to exogenous signals and regulating downstream target genes. Transcriptomic analysis revealed a total of 110 transcription factors among 807 significant DEGs under 6-BA treatment. TFs are encompassed in 26 families, including MYB (21), bHLH (17), AP2/ERF-ERF (15), and NAC (15) had the highest number of genes. Various TFs families have been identified to be responsible for gene regulation of cell elongation in response to different environmental signals; for example, MYB42 and MYB85 redundantly and negatively regulate hypocotyl cell elongation by mediating BR signaling in *Arabidopsis thaliana*, MYB42 transcription was suppressed by BR treatment and mutation of both MYB42 and MYB85 enhanced the dwarf phenotype of the BR receptor mutant bri1-5 (Zhuang et al., 2022). Additionally, MYBH is one of the molecular components that regulate hypocotyl elongation in response to darkness (Kwon et al., 2013), indicating that the large number of MYB TFs may regulate hypocotyl elongation under darkness in pine leaves. bHLH family contains the second-highest number of TFs, suggesting that bHLH possibly played a role to transfer external signals under 6-BA treatment. Two basic helix-loop-helix (bHLH) transcription factors (TFs), bHLH48 and bHLH60 (bHLH48/bHLH60), positively regulate hypocotyl elongation by interacting with PIF7 and enhancing its DNA binding affinity in *Arabidopsis thaliana* (Yang et al., 2021). bHLH48/bHLH60 may ensure delicate, fine-tuned control of PIF7 activity, thus allowing plants to respond precisely to the shade environment. We found that AP2/ERF-ERF and NAC family also has 15 TFs; in response to hormonal signals, *AtERF11* plays a dual role in promoting internode elongation by inhibiting ethylene biosynthesis and activating GA biosynthesis and signaling pathways (Zhou et al., 2016). ANAC102 and ATAF1 gene-knockout mutants exhibit elevated expression of both BAS1 and SOB7, expanded tissue-level accumulation of their protein products and reduced hypocotyl growth in response to exogenous BR treatments (Peng and Neff, 2021). Though the number of MYB, bHLH, AP2/ERFs and NAC studied in the hormones regulatory network in angiosperms, the molecular mechanisms underlying the large number of these transcription factor families in conifers in response to hormones stimulation and the formation of multifaceted regulation under dark conditions are still worth further investigation.

In cell elongation, especially hypocotyl elongation, the expression of downstream proteins regulated by transcription factors is critical to produce physiological and biochemical changes (Gendreau et al., 1997). A large number of growth hormone-related and cell wall-associated proteins, such as Small Auxin Up-Regulated enzymes (SAURs), expansins (EXPs), cell wall remodeling enzymes called xyloglucan endotransglucosylase/hydrolases (XTHs), and WALLS ARE THIN1-related enzymes (WAT1s), are involved in hypocotyl growth (Collett et al., 2000; Boron and Vissenberg, 2014; Du et al., 2020; Lin et al., 2021). In *Arabidopsis thaliana*, the SHORT-ROOT (SHR) transcription factor controls hypocotyl cell elongation *via* the transcriptional regulation of XTH18, XTH22, and XTH24 (Dhar et al., 2022). Other report also found that XTH proteins may play an important role in regulating cell wall extensibility and thus cell elongation in soybean hypocotyls (Wu et al., 2005). Furthermore, some reports in Arabidopsis have demonstrated that auxin-induced SMALL AUXIN UP RNA (SAUR) genes promote elongation growth and play a key role in PM H+-ATPase activation by inhibiting PP2C.D family protein phosphatases (Stamm and Kumar, 2013; Zhou et al., 2016; Spartz et al., 2017). Through transcriptome analysis and functional annotation, we found that a large number of these genes differentially expressed and their expression level was strongly repressed in *P. crassifolia* in response to 6-BA treatment. These types of genes are involved in the growth hormone signaling pathway in the regulation of cell elongation, suggesting that exogenous cytokinin signaling in *P. crassifolia* may interact with some nodes in the growth hormone signaling pathway to suppress the expression of growth hormone and cell wall-related genes, thereby inhibiting hypocotyl elongation. Another noteworthy is the strong upregulation of multiple cytokinin dehydrogenase genes under 6-BA treatment. Cytokinin oxidase/dehydrogenase (CKX) is the main enzyme for inactivating cytokinins by irreversibly cleaving their N6 side chains to generate adenine or adenosine. Directly or indirectly targeting CKX could influence cytokinin homeostasis (Schmuelling et al., 2003; Zhang et al., 2021). These results indicated that *P. crassifolia* used CTD to degrade cytokinin after the application of exogenous cytokinin and at the same time repressed the expression of growth auxin and wall-related genes, and the two pathways collectively inhibited the hypocotyl growth. In Arabidopsis, the inhibition of cytokinin on hypocotyl elongation appears to be mediated largely by the production of ethylene (Cary et al., 1995). This may imply that cytokinins are mutually coupled with other hormonal pathways to inhibit hypocotyl growth, while the main coupling hormones and molecular mechanisms are not the same in angiosperms and gymnosperms.

After we obtained a series of key transcription factors and downstream proteins, we predicted the interrelationships and co-expression networks among these genes based on gene expression trends, relative expression level validation and gene correlations. *PcbHLH149*, *PcMYB44*, *PcERF14*, *PcCTD1*, *PcCTD3*, *PcCTD6*, *PcXTH31*, *PcWAT1-1* and *PcSAUR15* were potential core genes involved in the inhibition of hypocotyl elongation under exogenous 6-BA treatment in *P. crassifolia*. (Wilson, 1964; Eklof and Brumer, 2010) XTH proteins are widely found in various plants tissues and cells and can modify the cellulose-xyloglucan complex structure of plant cell walls by catalyzing the breakage and reconnection of xyloglucan molecules to achieve cell wall remodeling (Wilson, 1964; Eklof and Brumer, 2010). XTHs are important for cell elongation in plants, however, their regulatory network remains unclear. Previous reports have demonstrated that the expression level change of XTH genes can influence hypocotyls elongation, for example, overexpression of *AtXTH18*, *AtXTH19*, and *AtXTH20* stimulated growth of hypocotyls in Arabidopsis (Miedes et al., 2013). Several XTH genes were strongly inhibited in their expression level by exogenous 6-BA treatment in *P. crassifolia*, indicating that XTH proteins are able to respond to cytokinin signals in *P. crassifolia*. *PcXTH31* was a potential core XTH genes to regulate the *P. crassifolia* cell wall structure hypocotyls in response to exogenous 6-BA. WAT1 was expressed in Arabidopsis all tissues and organs, with the highest expression in stems and hypocotyls, and may play an important regulatory role in plant fiber secondary wall formation through the regulation of genes encoding the secondary wall-associated NAC structural domain protein SND1 and the NAC secondary wall thickening promoter NST1 (Mitsuda et al., 2007; Ranocha et al., 2010). Besides, the BR-SlBZR1/2-WAT1 signalling network contributes to the high level of auxin signalling in the vascular cambium for secondary growth in *Solanum lycopersicum* (Lee et al., 2021). These suggesting that WAT1 is able to respond to hormonal signals involved in regulating the formation and development of secondary walls and plays an important role in hypocotyl growth. *PcXTH31*, *PcWAT1-1* and *PcSAUR15* play an important role in regulating hypocotyl growth in response to upstream hormone signals to regulate the cell wall structure and can be used as core proteins for subsequent studies.

In the regulatory network, not only downstream proteins are required to function, but also transcription factors are required to signal and regulate the expression of downstream genes. We found 10 closely associated core TFs connected with the 6-BA treatment. *PcbHLH149* was a hub gene in all the different co-expression network analysis (**Figure 6**). bHLH transcription factor family has been widely reported to regulate cell elongation in angiosperms, and different transcription factors in this family play different roles in cell elongation. Such as two bHLH transcription factors, LP1 and LP2 proteins, could directly bind to the promoters of Longifolia1 (LNG1) and LNG2 to activate the expression of these cell elongation related genes in Arabidopsis (Lu et al., 2021). Three bHLH proteins, PACLOBTRAZOL RESISTANCE1 (PRE1), Cryptochrome Interacting Basic Helix-loop-helix 5 (CIB5), and Arabidopsis ILI1 binding bHLH1 (IBH1) form a triantagonistic system that antagonistically regulates cell elongation in a competitive manner (Hou et al., 2022). Therefore, *PcbHLH149* is likely to be a candidate transcription factor for the regulation of downstream core proteins (*PcXTH31*, *PcWAT1-1* and *PcSAUR15*). There may be a multi-layered regulatory network between these screened transcription factors and downstream proteins to regulate hypocotyl elongation. The inhibitory effect on hypocotyl growth was achieved by responding to and transmitting exogenous cytokinin signals, increasing the expression of CTD to catabolize cytokinin, and inhibiting the expression of XTH, WAT1, SAUR and other proteins to prevent the relaxation of cell wall structures and cell elongation.

## Data availability statement

The datasets presented in this study can be found in online repositories. The names of the repository/repositories and accession number(s) can be found below: https://www.ncbi.nlm.nih.gov/, PRJNA895003.

## Author contributions

HL and CZ: Conceptualization, methodology, investigation, visualization, writing - original draft, writing - review & editing. ZN: Sample treatments and collection. WL and YE-K: Conceived the study and revised the manuscript. All authors contributed to the article and approved the submitted version.

## Supplementary material

Supplementary Figure 1 Effect of 100 μ mol L^-1^ ACC and 6-BA on hypocotyl elongation in *P. crassifolia* hypocotyls. **(A)** Phenotypes of P. crassifolia hypocotylstreated with the same concentration of ACC and 6-BA under dark. **(B)** Cloud and rain plots of hypocotyl length statistics of P. crassifolia treated with the same concentration of ACC and 6-BA under dark

## References

[B1] Binder B. M. (2020). Ethylene signaling in plants. J. Of Biol. Chem. 295, 7710–7725. doi: 10.1074/jbc.REV120.010854 32332098 PMC7261785

[B2] Boron A. K. Vissenberg K. (2014). *Arabidopsis thaliana* hypocotyl, a model to identify and study control mechanisms of cellular expansion. Plant Cell Rep. 33, 697–706. doi: 10.1007/s00299-014-1591-x 24633990

[B3] Cary A. J. Liu W. Howell S. H. (1995). Cytokinin action is coupled to ethylene in its effects on the inhibition of root and hypocotyl elongation in *Arabidopsis thaliana* seedlings. Plant Physiol. 107, 1075–1082. doi: 10.1104/pp.107.4.1075 7770519 PMC157239

[B4] Cheadle C. Vawter M. P. Freed W. J. Becker K. G. (2003). Analysis of microarray data using z score transformation. J. Mol. Diagnostics 5, 73–81. doi: 10.1016/S1525-1578(10)60455-2 PMC1907322 12707371

[B5] Collett C. E. Harberd N. P. Leyser O. (2000). Hormonal interactions in the control of arabidopsis hypocotyl elongation. Plant Physiol. (Bethesda) 124, 553–561. doi: 10.1104/pp.124.2.553 PMC59162 11027706

[B6] Dan H. Imaseki H. Wasteneys G. O. Kazama H. (2003). Ethylene stimulates endoreduplication but inhibits cytokinesis in cucumber hypocotyl epidermis. Plant Physiol. 133, 1726–1731. doi: 10.1104/pp.103.025783 14645725 PMC300727

[B7] Dhar S. Kim J. Yoon E. K. Jang S. Ko K. Lim J. (2022). SHORT-ROOT controls cell elongation in the etiolated arabidopsis hypocotyl. Molecules Cells 45, 243–256. doi: 10.14348/molcells.2021.5008 35249891 PMC9001151

[B8] Du M. Spalding E. P. Gray W. M. (2020). Rapid auxin-mediated cell expansion. Annu. Rev. Plant Biol. 71, 379–402. doi: 10.1146/annurev-arplant-073019-025907 32131604 PMC7733314

[B9] Eklof J. M. Brumer H. (2010). The XTH gene family: An update on enzyme structure, function, and phylogeny in xyloglucan remodeling. Plant Physiol. 153, 456–466. doi: 10.1104/pp.110.156844 20421457 PMC2879796

[B10] Fan Y. J. Grebenc T. Wei J. Zhao Y. L. Yan W. Wang L. B. (2016). Association of ectomycorrhizal fungi with *Picea crassifolia* (Pinaceae, piceoidae) from high-altitude stands in mount helan nature reserve, China. Genet. Mol. Res. 15, 15038604. doi: 10.4238/gmr.15038604 27706692

[B11] Farjon A. (2018). Conifers of the world. Kew Bull. 73, 8. doi: 10.1007/s12225-018-9738-5

[B12] Feller A. Machemer K. Braun E. L. Grotewold E. (2011). Evolutionary and comparative analysis of MYB and bHLH plant transcription factors. Plant J. 66, 94–116. doi: 10.1111/j.1365-313X.2010.04459.x 21443626

[B13] Feng S. Martinez C. Gusmaroli G. Wang Y. Zhou J. Wang F.. (2008). Coordinated regulation of *Arabidopsis thaliana* development by light and gibberellins. Nature 451, 475–479. doi: 10.1038/nature06448 18216856 PMC2562044

[B14] Franklin K. A. Lee S. H. Patel D. Kumar S. V. Spartz A. K. Gu C.. (2011). PHYTOCHROME-INTERACTING FACTOR 4 (PIF4) regulates auxin biosynthesis at high temperature. Proc. Natl. Acad. Sci. - PNAS 108, 20231–20235. doi: 10.1073/pnas.1110682108 22123947 PMC3250122

[B15] Gendreau E. Traas J. Desnos T. Grandjean O. Caboche M. Hofte H. (1997). Cellular basis of hypocotyl growth in *Arabidopsis thaliana* . Plant Physiol. 114, 295–305. doi: 10.1104/pp.114.1.295 9159952 PMC158305

[B16] Gray W. M. Ostin A. Sandberg G. Romano C. P. Estelle M. (1998). High temperature promotes auxin-mediated hypocotyl elongation in *Arabidopsis* . Proc. Of Natl. Acad. Of Sci. Of United States Of America 95, 7197–7202. doi: 10.1073/pnas.95.12.7197 PMC22781 9618562

[B17] Hao Y. Zong X. Ren P. Qian Y. Fu A. (2021). Basic helix-Loop-Helix (bHLH) transcription factors regulate a wide range of functions in arabidopsis. Int. J. Of Mol. Sci. 22, 7152. doi: 10.3390/ijms22137152 34281206 PMC8267941

[B18] Hou Q. Zhao W. Lu L. Wang L. Zhang T. Hu B.. (2022). Overexpression of HLH4 inhibits cell elongation and anthocyanin biosynthesis in *Arabidopsis thaliana* . Cells (Basel Switzerland) 11, 1087. doi: 10.3390/cells11071087 PMC8997993 35406652

[B19] Ince Y.Ç. Galvão V. C. (2021). Analysis of shade-induced hypocotyl elongation in arabidopsis. Methods Mol. Biol. 2297, 21–31. doi: 10.1007/978-1-0716-1370-2_3 33656666

[B20] Jiang H. Shui Z. Xu L. Yang Y. Li Y. Yuan X.. (2020). Gibberellins modulate shade-induced soybean hypocotyl elongation downstream of the mutual promotion of auxin and brassinosteroids. Plant Physiol. And Biochem. 150, 209–221. doi: 10.1016/j.plaphy.2020.02.042 32155449

[B21] Ju W. Y. Li J. X. Yu W. R. Zhang R. C. (2016). iGraph: an incremental data processing system for dynamic graph. Front. Comput. Sci. 10, 462–476. doi: 10.1007/s11704-016-5485-7

[B22] Kwon Y. Kim J. H. Nguyen H. N. Jikumaru Y. Kamiya Y. Hong S.W.. (2013). A novel *Arabidopsis* MYB-like transcription factor, MYBH, regulates hypocotyl elongation by enhancing auxin accumulation. J. Of Exp. Bot. 64, 3911–3922. doi: 10.1093/jxb/ert223 23888064 PMC3745742

[B23] Langmead B. Salzberg S. L. (2012). Fast gapped-read alignment with bowtie 2. Nat. Methods 9, 357–359. doi: 10.1038/nmeth.1923 22388286 PMC3322381

[B24] Lee J. Kim H. Park S. G. Hwang H. Yoo S.I. Bae W.. (2021). Brassinosteroid-BZR1/2-WAT1 module determines the high level of auxin signalling in vascular cambium during wood formation. New Phytol. 230, 1503–1516. doi: 10.1111/nph.17265 33570747

[B25] Li B. Dewey C. N. (2011). RSEM: accurate transcript quantification from RNA-seq data with or without a reference genome. BMC Bioinf. 12, 323. doi: 10.1186/1471-2105-12-323 PMC3163565 21816040

[B26] Li W. Godzik A. (2006). Cd-hit: a fast program for clustering and comparing large sets of protein or nucleotide sequences. Bioinformatics 22, 1658–1659. doi: 10.1093/bioinformatics/btl158 16731699

[B27] Lin W. Zhou X. Tang W. Takahashi K. Pan X. Dai J.. (2021). TMK-based cell-surface auxin signalling activates cell-wall acidification. Nat. (London) 599, 278–282. doi: 10.1038/s41586-021-03976-4 34707287 PMC8549421

[B28] Liu Y. Sun C. Li Q. Cai Q. Añel J. A. (2016). A *Picea crassifolia* tree-ring width-based temperature reconstruction for the mt. dongda region, Northwest China, and its relationship to Large-scale climate forcing. PloS One 2016, e160963. doi: 10.1371/journal.pone.0160963 PMC4979898 27509206

[B29] Liu H. Zhang Y. Lu S. Chen H. Wu J. Zhu X.. (2021). HsfA1d promotes hypocotyl elongation under chilling *via* enhancing expression of ribosomal protein genes in arabidopsis. New Phytol. 231, 646–660. doi: 10.1111/nph.17413 33893646

[B30] Lu R. Zhang J. Wu Y. W. Wang Y. Zhang J. Zheng Y.. (2021). bHLH transcription factors LP1 and LP2 regulate longitudinal cell elongation. Plant Physiol. 187, 2577–2591. doi: 10.1093/plphys/kiab387 34618066 PMC8644604

[B31] Lucas M. D. Daviere J. M. Rodriguez-Falcon M. Pontin M. Iglesias-Pedraz JM. Lorrain S.. (2008). A molecular framework for light and gibberellin control of cell elongation. NATURE 451, 480–484. doi: 10.1038/nature06520 18216857

[B32] Lucas M. Prat S. (2014). PIFs get BRright: PHYTOCHROME INTERACTING FACTORs as integrators of light and hormonal signals. New Phytol. 202, 1126–1141. doi: 10.1111/nph.12725 24571056

[B33] Mashiguchi K. Tanaka K. Sakai T. Sugawara S. Kawaide H. Natsume M.. (2011). The main auxin biosynthesis pathway in *Arabidopsis* . Proc. Natl. Acad. Sci. - PNAS 108, 18512–18517. doi: 10.1073/pnas.1108434108 22025724 PMC3215075

[B34] Meng L. Yang R. Abbott R. J. Miehe G. Hu T. Liu J. (2007). Mitochondrial and chloroplast phylogeography of *Picea crassifolia* kom. (Pinaceae) in the qinghai-Tibetan plateau and adjacent highlands: PHYLOGEOGRAPHY OF PICEA CRASSIFOLIA. Mol. Ecol. 16, 4128–4137. doi: 10.1111/j.1365-294X.2007.03459.x 17894761

[B35] Miedes E. Suslov D. Vandenbussche F. Kenobi K. Ivakov A. Van Der Straeten D.. (2013). Xyloglucan endotransglucosylase/hydrolase (XTH) overexpression affects growth and cell wall mechanics in etiolated *Arabidopsis* hypocotyls. J. Of Exp. Bot. 64, 2481–2497. doi: 10.1093/jxb/ert107 23585673

[B36] Mitsuda N. Iwase A. Yamamoto H. Yoshida M. Seki M. Shinozaki K.. (2007). NAC transcription factors, NST1 and NST3, are key regulators of the formation of secondary walls in woody tissues of *Arabidopsis* . Plant Cell 19, 270–280. doi: 10.1105/tpc.106.047043 17237351 PMC1820955

[B37] Nieto C. López-Salmerón V. Davière J. Prat S. (2015). ELF3-PIF4 interaction regulates plant growth independently of the evening complex. Curr. Biol. 25, 187–193. doi: 10.1016/j.cub.2014.10.070 25557667

[B38] Niu S. Li J. Bo W. Yang W. Zuccolo A. Giacomello C.. (2022). The Chinese pine genome and methylome unveil key features of conifer evolution. CELL 185, 204–217. doi: 10.1016/j.cell.2021.12.006 34965378

[B39] Oh E. Zhu J. Bai M. Arenhart R. A. Sun Y. Wang Z. (2014). Cell elongation is regulated through a central circuit of interacting transcription factors in the arabidopsis hypocotyl. eLife 3, e03031. doi: 10.7554/eLife.03031.025 24867218 PMC4075450

[B40] Peng H. Neff M. M. (2021). Two ATAF transcription factors ANAC102 and ATAF1 contribute to the suppression of cytochrome P450-mediated brassinosteroid catabolism in *Arabidopsis* . Physiol. Plantarum 172, 1493–1505. doi: 10.1111/ppl.13339 33491178

[B41] Ranocha P. Denance N. Vanholme R. Freydier A. Martinez Y. Hoffmann L.. (2010). *Walls are thin 1* (*WAT1*), an arabidopsis homolog of *Medicago truncatula NODULIN21*, is a tonoplast-localized protein required for secondary wall formation in fibers. Plant J. 63, 469–483. doi: 10.1111/j.1365-313X.2010.04256.x 20497379

[B42] Reed J. W. Wu M. F. Reeves P. H. Hodgens C. Yadav V. Hayes S.. (2018). Three auxin response factors promote hypocotyl elongation. Plant Physiol. (Bethesda) 178, 864–875. doi: 10.1104/pp.18.00718 PMC6181040 30139794

[B43] Schmuelling T. I. O. B. Werner T. Riefler M. Krupkova E. Bartrina Y. Manns I. (2003). Structure and function of cytokinin oxidase/dehydrogenase genes of maize, rice, *Arabidopsis* and other species. J. Of Plant Res. 116, 241–252. doi: 10.1007/s10265-003-0096-4 12721786

[B44] Seo D. H. Yoon G. M. (2019). Light-induced stabilization of ACS contributes to hypocotyl elongation during the dark-to-light transition in arabidopsis seedlings. Plant J. 98, 898–911. doi: 10.1111/tpj.14289 30776167

[B45] Sliwinska E. Bassel G. W. Bewley J. D. (2009). Germination of *Arabidopsis thaliana* seeds is not completed as a result of elongation of the radicle but of the adjacent transition zone and lower hypocotyl. J. Of Exp. Bot. 60, 3587–3594. doi: 10.1093/jxb/erp203 19620183

[B46] Smalle J. Haegman M. Kurepa J. Van Montagu M. Straeten D. V. (1997). Ethylene can stimulate *Arabidopsis* hypocotyl elongation in the light. Proc. Of Natl. Acad. Of Sci. Of United States Of America 94, 2756–2761. doi: 10.1073/pnas.94.6.2756 PMC20163 11038610

[B47] Smets R. Le J. Prinsen E. Verbelen J. P. Van Onckelen H. A. (2005). Cytokinin-induced hypocotyl elongation in light-grown *Arabidopsis* plants with inhibited ethylene action or indole-3-acetic acid transport. Planta 221, 39–47. doi: 10.1007/s00425-004-1421-4 15843964

[B48] Song J. Cao K. Hao Y. Song S. Su W. Liu H. (2019). Hypocotyl elongation is regulated by supplemental blue and red light in cucumber seedling. GENE 707, 117–125. doi: 10.1016/j.gene.2019.04.070 31034942

[B49] Spartz A. K. Lor V. S. Ren H. Olszewski N.E. Miller N.D. Wu G.. (2017). Constitutive expression of arabidopsis *SMALL AUXIN UP RNA19* (*SAUR19*) in tomato confers auxin-independent hypocotyl elongation. Plant Physiol. (Bethesda) 173, 1453–1462. doi: 10.1104/pp.16.01514 PMC5291034 27999086

[B50] Stamm P. Kumar P. P. (2013). Auxin and gibberellin responsive *Arabidopsis SMALL AUXIN UP RNA36* regulates hypocotyl elongation in the light. Plant Cell Rep. 32, 759–769. doi: 10.1007/s00299-013-1406-5 23503980

[B51] Sun X. Wang Y. Sui N. (2018). Transcriptional regulation of bHLH during plant response to stress. Biochem. And Biophys. Res. Commun. 503, 397–401. doi: 10.1016/j.bbrc.2018.07.123 30057319

[B52] Tang Y. Li M. Wang J. Pan Y. Wu F. X. (2015). CytoNCA: a cytoscape plugin for centrality analysis and evaluation of protein interaction networks. Biosystems 127, 67–72. doi: 10.1016/j.biosystems.2014.11.005 25451770

[B53] Thomas P. D. Kejariwal A. Campbell M. J. Mi H. Diemer K. Guo N.. (2003). PANTHER: a browsable database of gene products organized by biological function, using curated protein family and subfamily classification. Nucleic Acids Res. 31, 334–341. doi: 10.1093/nar/gkg115 12520017 PMC165562

[B54] Wang W. Mcdowell N. G. Liu X. Xu G. Wu G. Zeng X.. (2021). Contrasting growth responses of qilian juniper (*Sabina przewalskii*) and qinghai spruce (*Picea crassifolia*) to CO_2_ fertilization despite common water-use efficiency increases at the northeastern qinghai-Tibetan plateau. Tree Physiol. 41, 992–1003. doi: 10.1093/treephys/tpaa169 33367904

[B55] Wilson K. (1964). “The growth of plant cell walls,” in International review of cytology. Eds. Bourne G. H. Danielli J. F. (Academic Press), 1–49. 10.1016/s0074-7696(08)60404-0 5337215

[B56] Wu Y. Jeong B. R. Fry S. C. Boyer J. S. (2005). Change in XET activities, cell wall extensibility and hypocotyl elongation of soybean seedlings at low water potential. PLANTA 220, 593–601. doi: 10.1007/s00425-004-1369-4 15375660

[B57] Yang C. Huang S. Zeng Y. Liu C. Ma Q. Pruneda-Paz J.. (2021). Two bHLH transcription factors, bHLH48 and bHLH60, associate with phytochrome interacting factor 7 to regulate hypocotyl elongation in *Arabidopsis* . Cell Rep. (Cambridge) 35, 109054. doi: 10.1016/j.celrep.2021.109054 33951433

[B58] Yu Y. Wang J. Zhang Z. Quan R. Zhang H. Deng X. W.. (2013). Ethylene promotes hypocotyl growth and HY5 degradation by enhancing the movement of COP1 to the nucleus in the light. PloS Genet. 9, e1004025. doi: 10.1371/journal.pgen.1004025 24348273 PMC3861121

[B59] Zhang W. Peng K. Cui F. Wang D. Zhao J. Zhang Y.. (2021). Cytokinin oxidase/dehydrogenase OsCKX11 coordinates source and sink relationship in rice by simultaneous regulation of leaf senescence and grain number. Plant Biotechnol. J. 19, 335–350. doi: 10.1111/pbi.13467 33448635 PMC7868977

[B60] Zhong S. Shi H. Xue C. Wang L. Xi Y. Li J.. (2012). A molecular framework of light-controlled phytohormone action in *Arabidopsis* . Curr. Biol. 22, 1530–1535. doi: 10.1016/j.cub.2012.06.039 22818915 PMC4437768

[B61] Zhou X. Zhang Z. L. Park J. Tyler L. Yusuke J. Qiu K.. (2016). The ERF11 transcription factor promotes internode elongation by activating gibberellin biosynthesis and signaling. Plant Physiol. 171, 2760–2770. doi: 10.1104/pp.16.00154 27255484 PMC4972265

[B62] Zhuang Y. Lian W. Tang X. Qi G. Wang D. Chai G.. (2022). MYB42 inhibits hypocotyl cell elongation by coordinating brassinosteroid homeostasis and signalling in *Arabidopsis thaliana* . Ann. Of Bot. 129, 403–413. doi: 10.1093/aob/mcab152 34922335 PMC8944714

