# Supplementary figures and images for: Exogenous 6-BA inhibited hypocotyl elongation under darkness in Picea crassifolia Kom revealed by transcriptome profiling
Source: Front Plant Sci. 2023 Feb 27;14:1086879. doi: 10.3389/fpls.2023.1086879 (PMC10009258; doi:10.3389/fpls.2023.1086879)

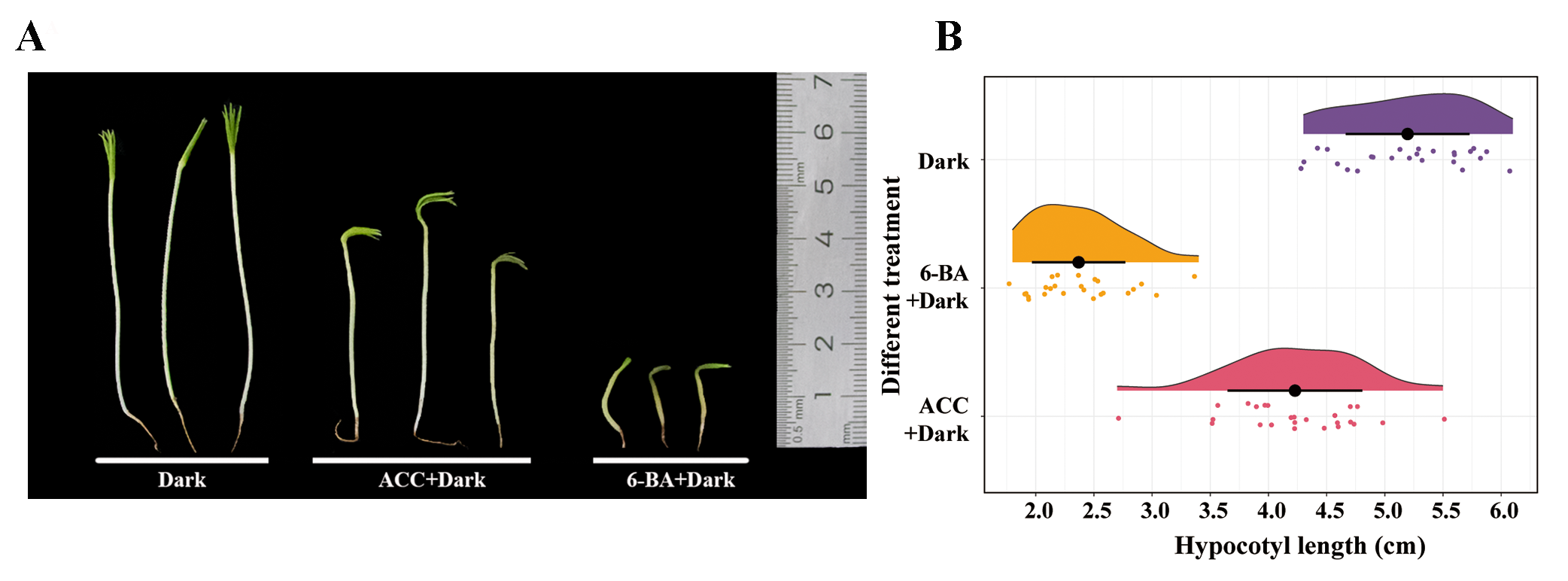

Supplement: Supplementary Figure 1 — Effect of 100 μ mol L-1 ACC and 6-BA on hypocotyl elongation in P. crassifolia hypocotyls. (A) Phenotypes of P. crassifolia hypocotylstreated with the same concentration of ACC and 6-BA under dark. (B) Cloud and rain plots of hypocotyl length statistics of P. crassifolia treated with the same concentration of ACC and 6-BA under dark [file Image_1.tif]
